# Pathogenicity and Epitope Characteristics Do Not Differ in IgG Subclass-Switched Anti-Desmoglein 3 IgG1 and IgG4 Autoantibodies in Pemphigus Vulgaris

**DOI:** 10.1371/journal.pone.0156800

**Published:** 2016-06-15

**Authors:** Agnes S. Lo, Xuming Mao, Eric M. Mukherjee, Christoph T. Ellebrecht, Xiaocong Yu, Marshall R. Posner, Aimee S. Payne, Lisa A. Cavacini

**Affiliations:** 1 Department of Medicine, Beth Israel Deaconess Medical Center, Dana-Farber Cancer Institute and Harvard Medical School, Boston, Massachusetts, United States of America; 2 Department of Dermatology, University of Pennsylvania, Philadelphia, Pennsylvania, United States of America; University of Missouri-Kansas City, UNITED STATES

## Abstract

Pemphigus vulgaris (PV) is characterized by IgG1 and IgG4 autoantibodies to desmoglein (Dsg) 3, causing suprabasal blistering of skin and mucous membranes. IgG4 is the dominant autoantibody subclass in PV and correlates with disease activity, whereas IgG1 can be associated with remittent disease. It is unknown if switching the same variable region between IgG4 and IgG1 directly impacts pathogenicity. Here, we tested whether three pathogenic PV monoclonal antibodies (mAbs) from three different patients demonstrate differences in antigen affinity, epitope specificity, or pathogenicity when expressed as IgG1 or IgG4. F706 anti-Dsg3 IgG4 and F779 anti-Dsg3 IgG1, previously isolated as heterohybridomas, and Px43, a monovalent anti-Dsg3/Dsg1 IgG antibody isolated by phage display, were subcloned to obtain paired sets of IgG1 and IgG4 mAbs. Using ELISA and cell surface staining assays, F706 and F779 demonstrated similar antigen binding affinities of IgG1 and IgG4, whereas Px43 showed 3- to 8-fold higher affinity of IgG4 versus IgG1 by ELISA, but identical binding affinities to human skin, perhaps due to targeting of a quaternary epitope best displayed in tissues. All 3 mAb pairs targeted the same extracellular cadherin (EC) domain on Dsg3, caused Dsg3 internalization in primary human keratinocytes, and caused suprabasal blisters in human skin at comparable doses. We conclude that switching IgG1 and IgG4 subclasses of pathogenic PV mAbs does not directly affect their antigen binding or pathogenic properties.

## Introduction

Pemphigus vulgaris is a potentially life threatening autoimmune blistering disease caused by IgG autoantibodies against Dsg3 +/- Dsg1, desmosomal adhesion proteins of keratinocytes [1]. In mucosal PV, autoantibodies react against Dsg3, which is the major Dsg isoform expressed in basal keratinocytes of the mucosa, leading to suprabasal blistering. In mucocutaneous PV, autoantibodies target both Dsg3 and Dsg1, causing suprabasal blisters in both mucosa and skin. Antibody cloning studies from PV patients have shown that a single mAb can bind Dsg3 only, or both Dsg3 and Dsg1 [2–4]. Epitope mapping studies indicate that autoantibodies from pemphigus patients with active disease bind near the N-terminus of Dsg3, where residues important for cis- and trans-adhesive interactions reside [4,5].

Although monovalent anti-Dsg antibody variable region fragments are sufficient to cause skin blisters in the absence of the constant region [6], preferential use of IgG subclasses has been identified in pemphigus patients. In active disease, anti-Dsg3 IgG4 autoantibodies are predominantly found in PV sera, followed by IgG1, and occasionally IgG2 and IgG3 [7]. In contrast, patients in remission and sometimes healthy relatives of PV patients can demonstrate anti-Dsg3 IgG1 [8,9]. Although some studies reported association of IgG1 with active disease and IgG4 with remittent disease [10], the majority of these early studies in the field suggested that IgG4 was pathogenic but IgG1 was not. Subsequently, heterohybridomas produced from PV patients have shown that pathogenic anti-Dsg3 antibodies can derive from either the IgG1 or IgG4 subclass [2,4,11].

While it is clear that IgG4 is associated with pathogenicity in PV, it is unknown whether the same variable region of a pathogenic IgG4 antibody, expressed on an IgG1 constant region, directly affects antibody function, for example antibody affinity, epitope binding characteristics, Dsg3 internalization, or pathogenicity. In the study presented here, we selected three representative pathogenic PV mAbs cloned from 3 different PV patients: F706, an anti-Dsg3 IgG4 isolated by heterohybridoma, F779, an anti-Dsg3 IgG1 isolated by heterohybridoma, and Px43, an anti-Dsg3/Dsg1 monovalent IgG antibody of unknown subclass isolated by phage display, and generated pairs of recombinant IgG1 and IgG4 antibodies expressing the identical variable region in order to assess the effects of the constant region on antibody function.

## Materials and Methods

### Human anti-Dsg3 mAb cloning

The three patients from whom the antibodies were cloned had active, extensive PV involving both mucosa and skin. The diagnosis of PV was confirmed by histology and immunofluorescence. All patients were off systemic therapies at the time of blood draw. F706 IgG4 and F779 IgG1 were isolated from two different patients by heterohybridoma and their desmoglein binding specificities were previously described [11,12]. Px43 was isolated from a third PV patient by phage display [3].

### Recombinant mAb subcloning, expression, and purification

Primers were designed to amplify the F706 and F779 heavy chain (HC) and light chain (LC) variable domains for subcloning into the pHC or pLC vector [13,14]. Primer sequences were as follows (restriction sites underlined):

5’ F706HC NheI: TCTAGCTAGCCGCCACCATGGACTGGACCTGGAGGGTCTT

3’ F706HC HindIII: TAGGGCAAGCTTGCTGAGGAGACGGTGACCAGG

5’ F706LC NheI: TCTAGCTAGCCGCCACCATGAGGCTCCTTGCTCAGCT

3’ F706LC NotI: TAGGGCGCGGCCGCAGTTCGTTTGATTTCCACCTT

5’ F779HC NheI: TCTAGCTAGCCGCCACCATGGACTGGACCTGGAGGGTCTT

3’ F779HC HindIII: TAGGGCAAGCTTGCTGAGGAGACGGTGACCAGG

5’ F779LC NheI: TCTAGCTAGCCGCCACCATGAGGCTCCTTGCTCAGCT

3’ F779LC NotI: TAGGGCGCGGCCGCAGTTCGTTTGATCTCCAGCTT

Recombinant DNA plasmids were co-transfected into CHO-K1 cell lines using Lipofectamine 2000 (Invitrogen, Grand Island, NY), and transfectants were cloned by limiting dilution in selective media (RPMI containing 10% fetal bovine serum, 10 μg/mL puromycin, 800 μg/mL G418) to establish permanent cell lines. The antibody supernatant was collected and purified by affinity chromatography on a Sepharose protein G column with acid glycine elution. F71 IgG1 mAb (targeting an unknown antigen) and anti-HIV b12 IgG4 mAb were used as isotype antibody controls for some *in vitro* experiments. Px43 IgG1 and IgG4 were produced as described [15,16].

### Epitope mapping

For epitope mapping, extracellular full length and domain-swapped Dsg1 and Dsg3 molecules were produced in baculovirus and the epitope determined by immunoprecipitation-immunoblot as previously described [17]. For epitope mapping of Px43, Dsg3-Dsg2 domain swapped molecules were used to identify targeted epitopes using immunoprecipitation-immunoblot as described [18]. All epitope mapping vectors were generously provided by Masayuki Amagai.

### Antibody ELISA

Dsg1 or Dsg3 ELISA (MBL International, Woburn, MA or Euroimmun, Luebeck, Germany) was used to measure relative binding affinities of IgG subclasses. Serial dilutions of IgG1 and IgG4 antibodies were incubated with Dsg1 or Dsg3 coated plates for 1 hour at room temperature using kit sample buffer or TBS buffer containing 1mM CaCl_2_ and 5% dry milk. After washing, bound antibody was detected using HRP-conjugated mouse monoclonal anti-human IgG F(ab')_2_ (MBL International, Woburn, MA) or mouse monoclonal anti-human lambda (clone JDC-12, Abcam, Cambridge, MA). After 1 hour incubation at room temperature, plates were washed, developed with 3,3’,5,5’ tetramethylbenzidine substrate, and optical density determined at 450 or 490 nm using an automated plate reader.

Relative affinity was determined using regression analysis of double-reciprocal plots of optical density versus antibody concentration. The relative constant was calculated as (1/y intercept)*slope.

### Direct and indirect immunofluorescence

Direct immunofluorescence (DIF) was performed on frozen human skin sections after mAb injection. Bound antibody in human skin was detected with Alexa Fluor 594 anti-human IgG (Invitrogen, Grand Island, NY) in TBS buffer containing 1mM CaCl_2_ and 1% BSA and visualized using an Olympus BX61 microscope.

Indirect immunofluorescence (IIF) was performed by incubating serial dilutions of IgG1 or IgG4 mAbs on frozen normal human skin sections (obtained through the Penn Skin Disease Research Center) in TBS buffer containing 1mM CaCl_2_ and 1% BSA. Bound antibody was subsequently detected with FITC-conjugated anti-human lambda light chain (clone JDC-12, Abcam, Cambridge, MA) and visualized using an Olympus BX61 microscope. The titer is reported as the last dilution at which epidermal cell surface staining is clearly positive.

### Immunoreactivity using live cell staining ELISA

Human oropharynx epithelial tumor cell line, UM-SCC-47 (SCC-47), was obtained from Dr. Thomas Carey, University of Michigan. Cell line was grown in 10% FBS-DMEM medium containing 10% heat inactivated FBS, 2 mM L-glutamine, 0.1 mM sodium pyruvate, 0.1 mM non-essential amino acids, and 10 μg/ml gentamicin. A live cell based ELISA was employed using SCC-47 cells seeded in a 96-well flat bottom plate for two days. Serial dilutions of PV IgG1 and IgG4 mAbs and isotype control antibodies were added to the cells for one hour. After washing, bound antibody was detected using biotin-conjugated goat anti-kappa chain antibody (1:5000) (Southern Biotech, Birmingham, AL) for one hour. The plate was washed and HRP-conjugated streptavidin (1:10,000) (SouthernBiotech) was added and incubated for 45 minutes. After a final wash, o-phenylenediamine (OPD) substrate (Sigma, St. Louis, MO) was added, and the plate was incubated for 20 min and read at 490nm.

### Human skin pathogenicity assay

Fresh normal human skin specimens were obtained from the Penn Skin Disease Research Center. The protocol was reviewed by the University of Pennsylvania Institutional Review Board, who conferred a human subjects research exemption, since the samples were obtained for clinical purposes, otherwise discarded, and de-identified, and therefore did not qualify as human subjects research. Skin was trimmed of fat and cut into 5 mm sections. 25–50 μg of purified isotype mAb, with or without 100 ng of *Staphylococcus aureus* exfoliative toxin A (ETA, Toxin Technology, Inc., Sarasota, FL) to inactivate Dsg1, was injected into skin sections, followed by incubation of skin pieces on transwell inserts (Corning, Lowell, MA), with defined keratinocyte serum free media (Invitrogen, Grand Island, NY) containing 1.2 mM CaCl_2_ in the outer compartment. Skin was harvested at 16–24 hours for DIF by embedding in OCT compound (TissueTek, Sakura, Netherlands) and for histology by fixation in 10% phosphate buffered formalin.

Primary human keratinocytes obtained from the Penn Skin Disease Research Center were treated with 50 μg/ml anti-Dsg3 mAbs for the indicated amount of time during calcium-induced (0.07 mM shift to 1.2 mM calcium) desmosome assembly as described [19]. At 8 hours, cells were double-labeled with Alexa Fluor 594 anti-human IgG and mouse anti-human Dsg3 (5G11) followed by Alexa Fluor 488 anti-mouse IgG, and nuclei were counter-stained with DAPI.

## Results

### IgG1 and IgG4 variants of pathogenic PV mAbs recognize the same antigenic epitopes

Because the hinge region of IgG1 differs from IgG4, creating differences in the orientation of the variable regions [20], Dsg3 affinity, epitope specificity, or pathogenicity could be affected by the presence of the IgG1 versus IgG4 Fc. To explore this further, IgG1 and IgG4 variants of pathogenic PV mAbs, bearing the identical variable region, were generated.

**Fig 1:**
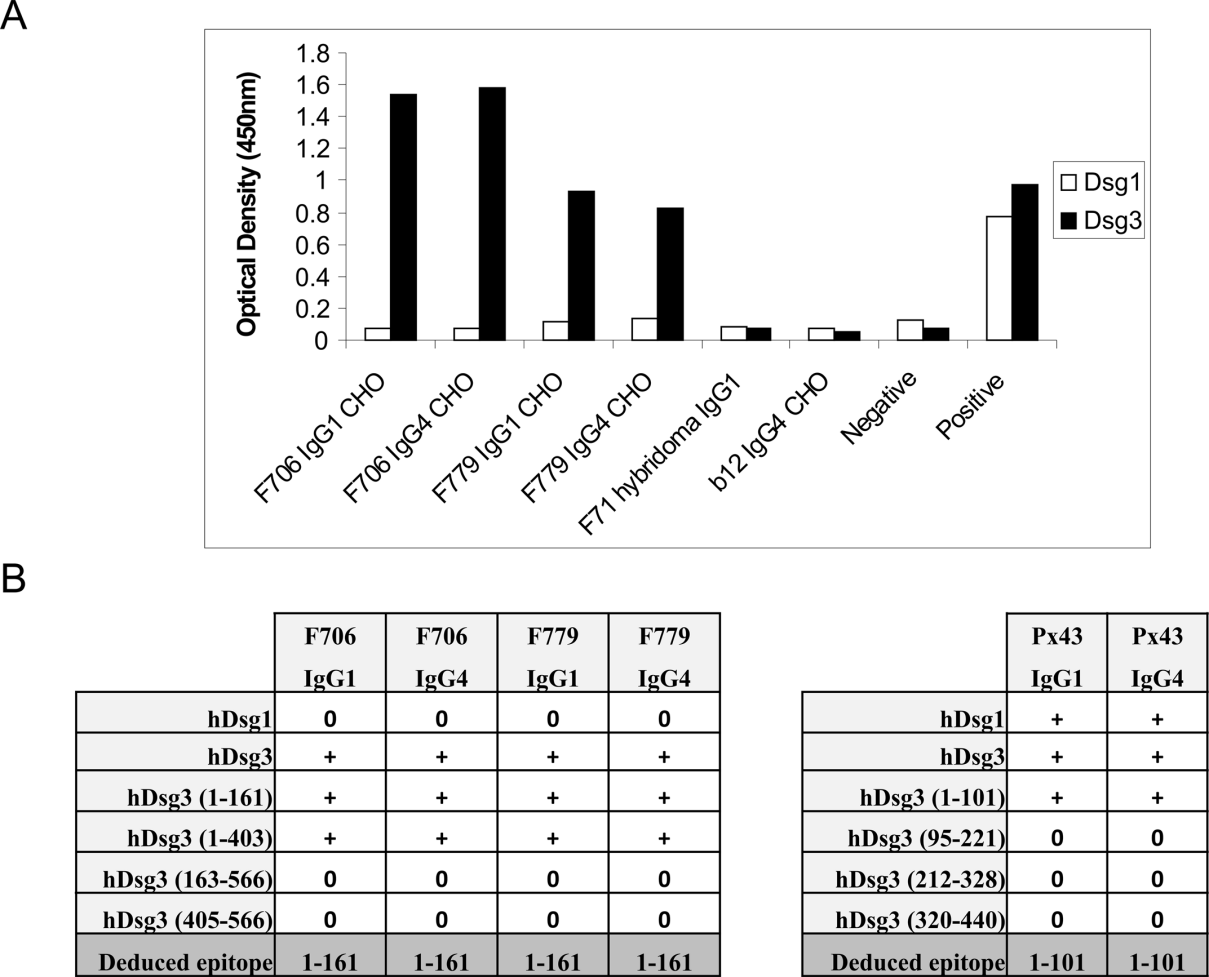
IgG1 and IgG4 PV mAbs target epitopes within the same Dsg3 extracellular domains. (A) F706 and F779 mAbs bind desmoglein 3 (Dsg3) but not Dsg1. Subclass-switched anti-human Dsg3 F706 and F779 IgG1 and IgG4 were tested by ELISA for binding to Dsg3 and Dsg1. Negative and positive controls were provided by the manufacturer. F71 IgG1 and b12 IgG4 antibodies served as isotype controls. (B) Deduced epitopes of IgG1 and IgG4 variants of PV mAbs show that mAb pairs recognize epitopes within the same Dsg3 extracellular domains.

To determine whether the IgG1 or IgG4 constant region affects epitope specificity, we first tested F706 and F779 IgG1 and IgG4 mAbs against recombinant purified Dsg1 and Dsg3 antigens. ELISA showed that F706 and F779 IgG1 and IgG4 mAbs specifically bound to Dsg3 but not Dsg1 (Fig 1A), indicating that the switch of constant region from IgG1 to IgG4 or from IgG4 to IgG1 in these pathogenic antibodies did not affect Dsg isoform specificity. To further define the epitope specificities, we performed conformational epitope mapping of IgG1 and IgG4 mAbs using Dsg3 domain-swapped chimeric molecules. F706 and F779, both IgG1 and IgG4, mapped to the first 161 amino acids of Dsg3 (Fig 1B). Similarly, Px43 IgG1 and IgG4 mapped to the same epitope within the first 101 amino acids of Dsg3. Thus, class-switched IgG1 and IgG4 variants do not change the variable region epitope specificity.

### PV IgG1 versus IgG4 mAbs demonstrate similar antigen affinities

**Fig 2:**
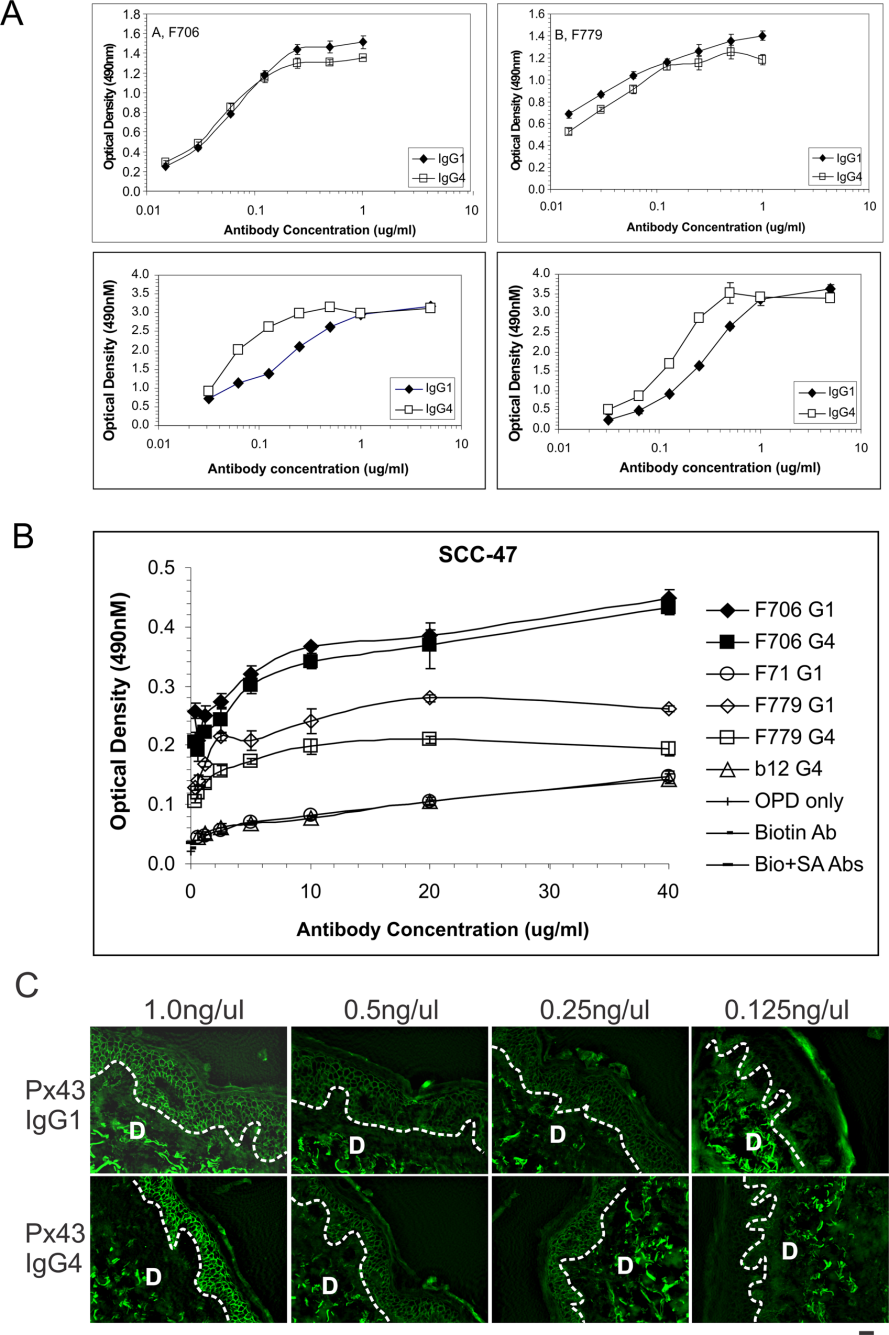
Immunoreactivity of anti-Dsg3 PV mAbs by ELISA and IIF. (A) Serial dilutions of recombinant human IgG1 (♦) or IgG4 (□) variants of mAbs (F706 against Dsg3 in upper left, F779 against Dsg3 in upper right, Px43 against Dsg3 in lower left and Px43 against Dsg1 in lower right) were incubated with Dsg3- coated ELISA plates. Bound antibody was detected using HRP conjugated mouse anti-human (H+L) Ab. Results are the mean of duplicate wells and are representative of 2–3 independent experiments. The relative affinity of each mAb was calculated by Lineweaver-Burk plot. (B) IgG1 and IgG4 variants of F706 and F779 mAbs show comparable immunoreactivity to the Dsg3+ human oropharynx tumor cell line SCC-47. F71 IgG1 and b12 IgG4 served as isotype controls. Other negative controls included HRP substrate OPD added alone or with secondary reagents only (secondary (2nd) biotinylated anti-human Fc Ab (Biotin Ab) or 2nd Ab with HRP-conjugated streptavidin Ab (Bio + SA Abs) in absence of tested PV mAbs. OPD: o-phenylenediamine dihydrochloride. (C) Px43 IgG1 and IgG4 demonstrate different relative affinities by ELISA but comparable affinity by IIF binding of human skin. D: dermal part of skin. Results are representative of 1–2 experiments.

To determine whether IgG1 or IgG4 constant regions affect antigen affinity, we evaluated IgG1 and IgG4 variants of pathogenic PV mAbs by ELISA and keratinocyte cell surface staining. IgG1 and IgG4 variants of F706 and F779 show a similar level of binding to Dsg3 by ELISA (Fig 2A). The apparent affinity of F706 IgG1 and IgG4 mAbs for Dsg3 is 0.10 μM and 0.067 μM, respectively, and 0.015 μM or 0.021 μM for F779 IgG1 or IgG4, respectively. Consistent with these data, binding isotherms of F706 and F779 IgG1 and IgG4 mAbs to Dsg3+ human oropharynx keratinocyte tumor cells by immunolabeling are similar (Fig 2B). Px43 IgG1 demonstrates 8-fold and 3-fold lower affinity for Dsg3 and Dsg1 than Px43 IgG4 by ELISA (Fig 2A); however their human skin IIF titers are similar at 0.25–0.5 ng/ μL (Fig 2C).

### PV mAb IgG1 versus IgG4 demonstrate comparable pathogenicity

**Fig 3:**
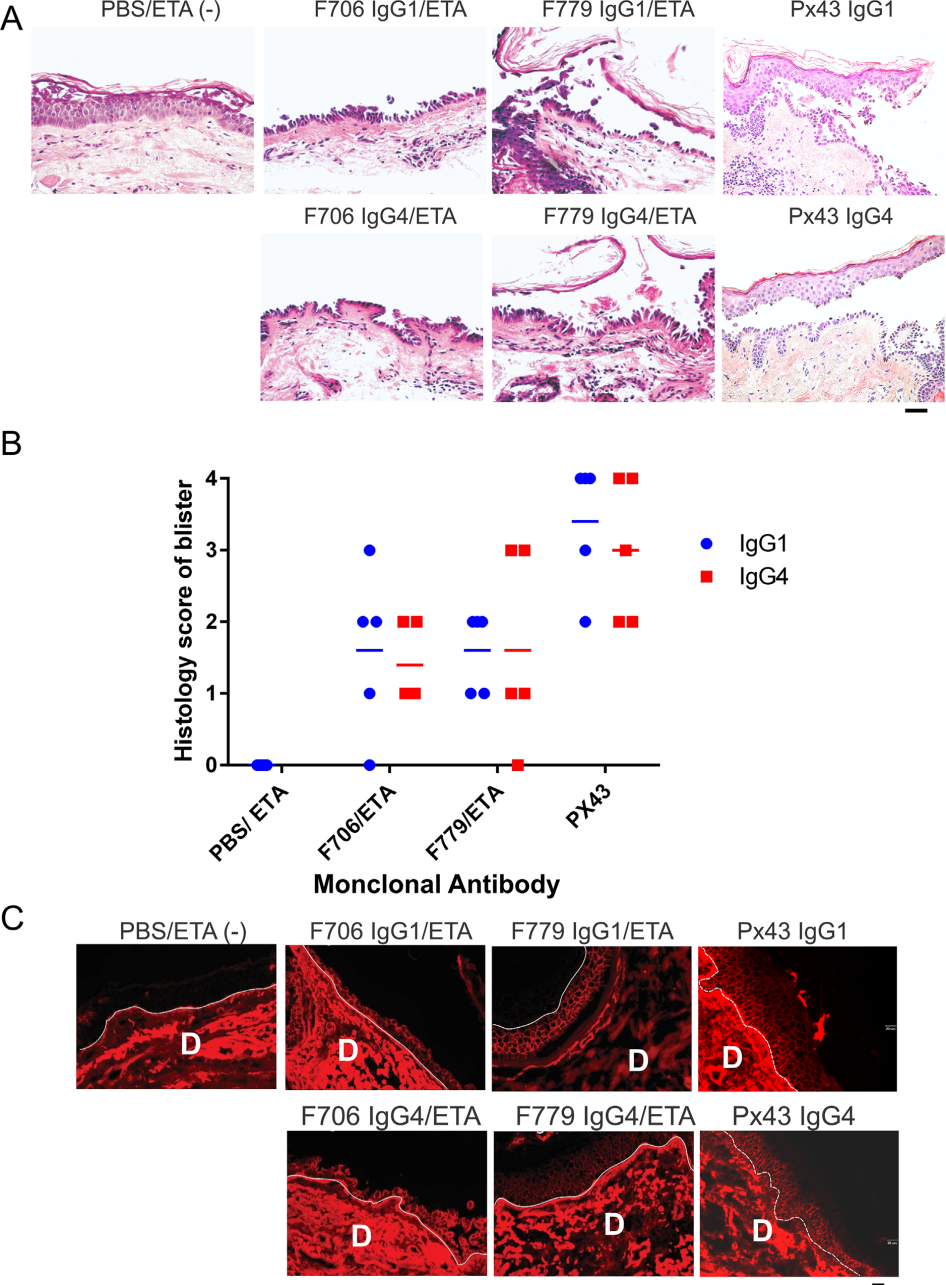
IgG1 and IgG4 variants of anti-Dsg3 PV mAbs are pathogenic and cause suprabasal blisters in human skin. (A) 25 μg anti-Dsg3 PV IgG1 or IgG4 was injected ex vivo into human skin sections and incubated for 18 hours prior to harvest for histologic analysis. IgG1 and IgG4 of three anti-Dsg3 PV mAbs, F706, F779 and Px43 induced suprabasal blisters in human skin. PBS/ETA served as negative control. Scale bar, 100 microns. (B) The extent of histologic blistering caused by IgG1 and IgG4 mAb variants is comparable. (C) DIF staining of human skin with PV isotype mAbs illustrates cell surface binding in skin epidermis following injection of mAbs. D: dermal part of skin. Scale bar, 20 microns.

We next investigated the pathogenicity of IgG1 and IgG4 recombinant mAbs, to determine whether IgG subclass could play a role in modulating the pathogenic effects of Dsg3-reactive variable regions. In skin, Dsg1 can provide compensatory adhesion if Dsg3 is inactivated [21,22]. Consequently, human skin assays to determine pathogenicity of anti-Dsg3 mAbs require inactivation of Dsg1, for example by co-injection of ETA, which specifically cleaves Dsg1 [23]. We injected 25–50 μg of anti-Dsg3 IgG1 or IgG4 mAbs, plus or minus ETA as required, into human skin sections, followed by incubation for 16–24 hours prior to harvest for immunofluorescence and histology. F706, F779, and Px43 all caused suprabasal blisters at comparable doses of IgG1 and IgG4 (Fig 3A). A similar extent of blistering was observed at both 25 μg and 50 μg doses (Fig 3B). DIF of injected skin sections showed comparable binding of PV IgG to the cell surface of keratinocytes (Fig 3C).

**Fig 4:**
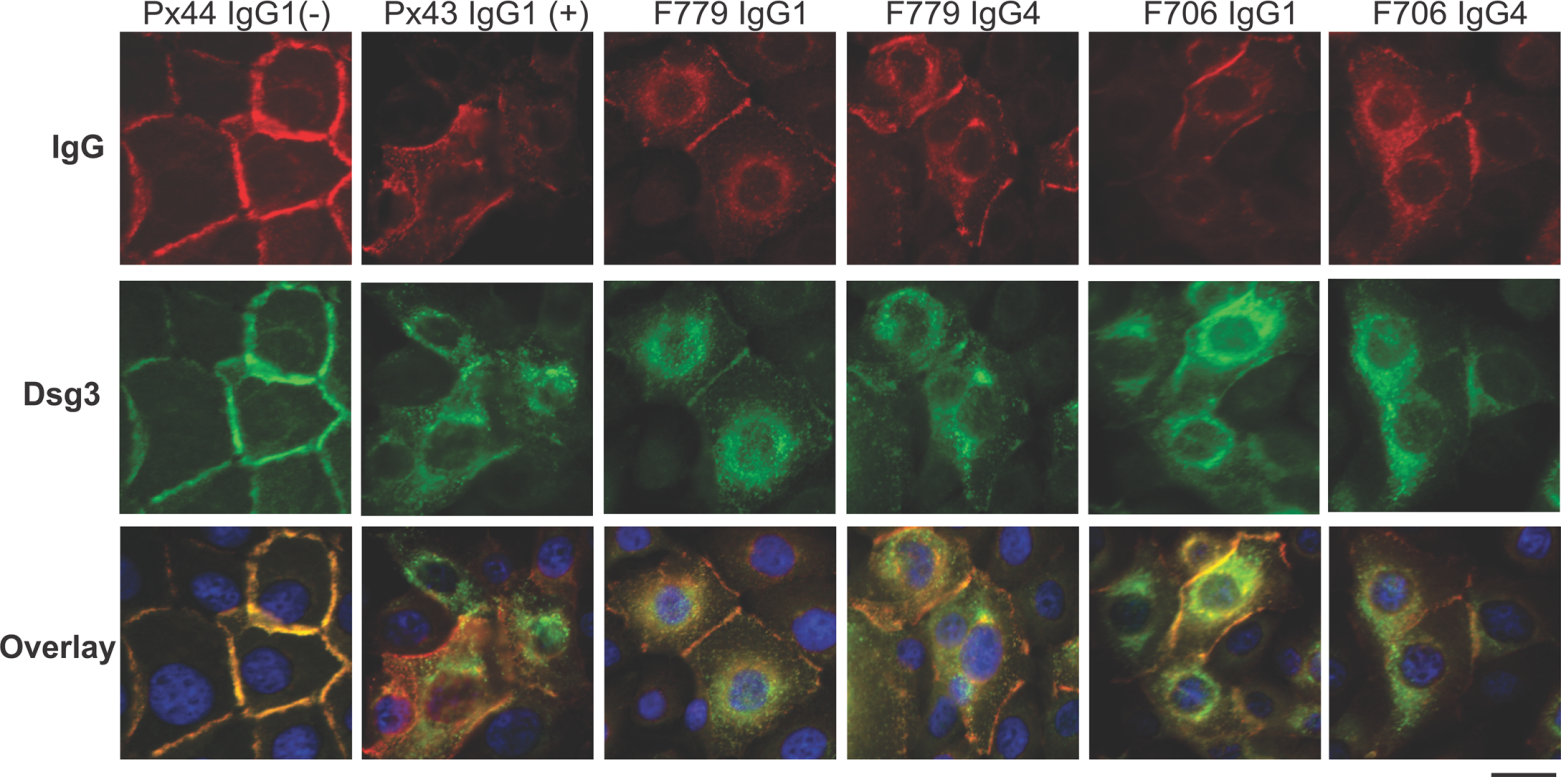
Pathogenic F706 and F779 IgG1 and IgG4 cause loss of cell surface Dsg3. Primary human keratinocytes were incubated for 8 hours with 50 μg/mL F706/F779 IgG1 or IgG4. Cells were prepared for IF staining using anti-human IgG or anti-Dsg3 (5G11) primary antibodies. Px44 negative and Px43 positive control antibodies were previously characterized [22]. Scale bar, 20 microns. Results are representative of two independent experiments.

Previously, pathogenic but not non-pathogenic human PV mAbs were shown to cause Dsg3 internalization in primary human keratinocytes [19]. Consistent with these studies, primary human keratinocytes treated with F706, F779, or Px43 IgG1 and IgG4 exhibited depletion of cell surface Dsg3 within 4 hours after antibody treatment, concomitant with an increase in cytoplasmic vesicular staining of Dsg3 (Fig 4). Thus, IgG1 and IgG4 variants of F706, F779, and Px43 comparably induce loss of cell surface Dsg3 in primary human epidermal keratinocytes and suprabasal blisters in human skin.

## Discussion

This study demonstrates that epitope specificity, antigen affinity, and pathogenicity of PV mAbs are not directly affected by the presence of the IgG1 versus IgG4 constant region. Although our conclusions are limited by the study of only three mAbs, these mAbs were cloned from 3 different patients and represent three distinct types of pathogenic PV mAbs that have been described in the literature, including anti-Dsg3 IgG1, anti-Dsg3 IgG4, and a cross-reactive anti-Dsg3/Dsg1 IgG.

All PV mAbs tested in this study bind the Dsg3 EC1-2 domains that are crucial for cis- and trans-adhesive interactions of the Dsgs. This result agrees with a previous large scale mapping study of PV sera in which 91% of PV sera mapped to the Dsg3 EC1 domain [18].

Although F706, F779, and Px43 all demonstrated comparable antigen affinity of IgG1 and IgG4 variants by ELISA and/or IIF staining, Px43 IgG1 and IgG4 were notable for demonstrating differing results by ELISA and IIF. Px43 was previously shown to recognize only mature conformational Dsg3 [24] and by surface plasmon resonance binds Dsg3 with complex kinetics that best fit to a conformational change model [11]. It is possible that Px43 binds a quaternary epitope in Dsg3 and Dsg1 that is predominant in native human skin but may be differentially displayed in ELISA, which may account for the difference in relative affinities observed between IgG1 and IgG4 variants. Nevertheless, these results indicate that the binding affinity of Px43 variable regions to Dsg3 in human skin is not significantly altered by the presence of the IgG1 versus IgG4 constant region.

No change of pathogenicity among PV IgG1 and IgG4 mAb variants was observed, in regard to Dsg3 internalization or the ability to cause suprabasal blisters in human skin. These data are consistent with the finding that the constant region is not required for autoantibody pathogenicity in PV [6] or for Dsg3 internalization after monovalent PV mAb binding [19]. Our data now further indicate that the IgG1 versus IgG4 constant region does not modulate the functional effects of pathogenic PV mAbs in regard to either Dsg3 internalization or suprabasal blister induction.

With no difference in pathogenicity, the question arises as to why IgG4 is preferentially associated with active disease. IgG4 is typically observed in conditions of chronic antigen stimulation, such as in individuals undergoing allergic desensitization or beekeepers [20]. Class switch recombination is a complex process regulated by cytokines [25]. IL-4 has a central role in stimulating antibody class switch [26]. CD40 and IL-4 can initiate class switch by inducing enzymes and other transcripts required for site-specific DNA recombination. IgG1 is the most abundant serum IgG subclass, in part due to its proximal location within the constant region locus. Repeated antigenic exposure can encourage subsequent isotype switching. IL-4 and IL-13 promote isotype switching first to IgG4 and subsequently to IgE [27], whereas IL-10 and IL-21 potentiates IL-4-induced switching to IgG4 over IgE [28,29]. Upregulation of the Th2 cytokines IL-4, IL-5, IL-10, and IL-13 has been described in pemphigus patients [30,31]. These cytokines would promote an IgG4>IgG1 serum antibody profile, coinciding with the spectrum of observed autoantibody isotypes in patients with active disease. A deeper understanding of how pathogenic variable regions segregate among the IgG subclasses would require isotype-specific antibody cloning.

In summary, we have found that subclass-switching between IgG1 and IgG4 has no significant effect on epitope specificity, antigen binding affinity, or pathogenicity in 3 human PV mAbs isolated from 3 different PV patients. Although limited by the study of only three antibodies, as well as the study of only pathogenic antibodies and not non-pathogenic antibodies, this study provides the first direct evidence that the immunochemical and pathogenic properties of PV pathogenic variable regions are not significantly modulated by antibody subclass, supporting the conclusion that the antibody variable region is most important for determining pathogenicity in PV. Our data also suggest that pathogenic PV mAbs cannot be engineered to be non-pathogenic agents for drug delivery based on modulation of the constant region. Instead, only antibodies with non-pathogenic anti-Dsg3 variable regions should be used as a tool for targeting drug delivery, for example to Dsg3+ head and neck squamous cell carcinomas [32] or inflammatory skin conditions [33]. Further research may shed light on potential diagnostic and therapeutic uses of human anti-Dsg3 mAbs.

## Abbreviations

PV: pemphigus vulgaris
Dsg: desmoglein
mAb: monoclonal antibody
DIF: direct immunofluorescence
IIF: indirect immunofluorescence
OPD: o-phenylenediamine dihydrochloride
ETA: *Staphylococcus aureus* exfoliative toxin A
EC: extracellular cadherin
HC: heavy chain
LC: light chain
